# Limited role of culture conversion for decision-making in individual patient care and for advancing novel regimens to confirmatory clinical trials

**DOI:** 10.1186/s12916-016-0565-y

**Published:** 2016-02-04

**Authors:** Patrick P. J. Phillips, Carl M. Mendel, Divan A. Burger, Angela Crook, Andrew J. Nunn, Rodney Dawson, Andreas H. Diacon, Stephen H. Gillespie

**Affiliations:** MRC Clinical Trials Unit at UCL, Aviation House, 125 Kingsway, London, WC2B 6NH UK; Global Alliance for TB Drug Development, New York, NY USA; Department of Mathematical Statistics and Actuarial Science, University of the Free State, Bloemfontein, South Africa; Division of Pulmonology and Department of Medicine, University of Cape Town Lung Institute, Mowbray, Cape Town South Africa; Division of Physiology, Department of Medical Biochemistry, Stellenbosch University, Tygerberg, Cape Town South Africa; TASK Applied Science, Bellville, Cape Town South Africa; School of Medicine, University of St Andrews, St Andrews, UK

**Keywords:** Tuberculosis, Clinical trials, Surrogate endpoints, Moxifloxacin

## Abstract

**Background:**

Despite recent increased clinical trials activity, no regimen has proved able to replace the standard 6-month regimen for drug-sensitive tuberculosis. Understanding the relationship between microbiological markers measured during treatment and long-term clinical outcomes is critical to evaluate their usefulness for decision-making for both individual patient care and for advancing novel regimens into time-consuming and expensive pivotal phase III trials.

**Methods:**

Using data from the randomized controlled phase III trial REMoxTB, we evaluated sputum-based markers of speed of clearance of bacilli: time to smear negative status; time to culture negative status on LJ or in MGIT; daily rate of change of log_10_(TTP) to day 56; and smear or culture results at weeks 6, 8 or 12; as individual- and trial-level surrogate endpoints for long-term clinical outcome.

**Results:**

Time to culture negative status on LJ or in MGIT, time to smear negative status and daily rate of change in log_10_(TTP) were each independent predictors of clinical outcome, adjusted for treatment (*p* <0.001). However, discrimination between low and high risk patients, as measured by the c-statistic, was modest and not much higher than the reference model adjusted for BMI, history of smoking, HIV status, cavitation, gender and MGIT TTP.

**Conclusions:**

Culture conversion during treatment for tuberculosis, however measured, has only a limited role in decision-making for advancing regimens into phase III trials or in predicting the outcome of treatment for individual patients. REMoxTB ClinicalTrials.gov number: NCT00864383.

## Background

The recent failure to reduce the duration of tuberculosis (TB) treatment from 6 to 4 months using fluoroquinolones in three major phase III trials [1–3] should prompt a review of how decisions are made to move novel regimens to pivotal phase III trials in the drug development pathway.

TB was declared a global emergency by the World Health Organization (WHO) as far back as 2003, with 9.0 million new cases and 1.5 million deaths worldwide from TB in 2013 [4]. It is widely recognized that new treatment regimens are urgently needed to end the TB epidemic [5]. New drugs and regimens are in the pipeline for drug-sensitive TB and multi-drug-resistant TB (MDR-TB) with a number of phase III trials for novel regimens starting over the next few years. Although there is a modest association between late culture conversion and poor outcomes for individual patients on standard treatment [6, 7], this relationship is unknown for other regimens. A better understanding of how the available microbiological markers measured during treatment relate to long-term clinical outcomes will enable improved decision-making for both individual patient care and moving regimens into time-consuming expensive pivotal phase III trials.

A surrogate endpoint is defined as “a laboratory measurement or a physical sign used as a substitute for a clinically meaningful endpoint. … Changes induced by a therapy on a surrogate endpoint are expected to reflect changes in a clinically meaningful endpoint” [8]. Although not usually a perfect surrogate, the primary efficacy endpoint of a phase II trial is chosen so that differences between interventions in the endpoint are expected to reflect differences between interventions in a more clinically meaningful phase III endpoint, irrespective of the interventions being compared. This is often described as trial-level surrogacy in contrast to individual-level surrogacy, which relates to the degree to which the results of an early outcome are predictive of the long-term clinical outcome in individual patients undergoing the same treatment.

Culture positivity on LJ solid media at either 2 or 3 months is not an acceptable surrogate endpoint for long-term clinical outcome [9–11], although it is the only marker that has undergone rigorous evaluation. The inherent lower statistical power of a dichotomous compared to a continuous endpoint means TB phase II trials are now rarely designed with these endpoints. Rather, time to culture conversion [12] or the slope of quantitative cultures on solid or liquid media over time [13, 14] are more commonly used as they permit smaller trials and are thought to be more reliable for comparing regimens by capturing an element of time on treatment. As an example, bedaquiline received accelerated approval by the US Food and Drug Administration (FDA) based on time to culture conversion as the primary efficacy measure [15]. Despite this, the place of these markers in regimen development has not yet been formally evaluated, mainly due to the paucity of data collected in the majority of previous TB phase III trials [16]. The REMoxTB trial was designed with weekly cultures during the first 8 weeks and monthly cultures to the end of treatment to allow for the evaluation of the role of various measures of bacillary clearance in response to treatment as individual-level and trial-level surrogates for long-term clinical outcome.

## Methods

Eligible patients in the REMoxTB trial were randomized to one of three daily regimens: a control regimen consisting of isoniazid and rifampicin for 6 months supplemented by pyrazinamide and ethambutol for the first 2 months; 4 months of rifampicin, moxifloxacin and isoniazid supplemented by pyrazinamide for the first 2 months (isoniazid arm); and 4 months of rifampicin and moxifloxacin supplemented by pyrazinamide and ethambutol for the first 2 months (ethambutol arm) as reported previously [1].

Sputum samples were taken for smear and culture weekly to 8 weeks during treatment, monthly thereafter to 6 months and 3-monthly thereafter to 18 months from randomization. All cultures were performed in parallel using LJ and MGIT and so time to culture negative status could be measured separately. Sputum was decontaminated with acetylcysteine–sodium hydroxide prior to culture and mycobacterial speciation was performed using the AccuProbe assay (Gen-Probe, San Diego, CA, USA). The REMoxTB laboratory and quality manuals are available on request.

Markers of speed of clearance of bacilli were determined as follows: 1) time to culture negative status on LJ or in MGIT; 2) rate of change of time to positivity on MGIT culture (TTP) over time; 3) time to smear negative status; 4) culture negative on LJ or in MGIT at 6, 8 or 12 weeks after randomization; and 5) smear negative at 6, 8 or 12 weeks after randomization. Time to culture negative status was defined as the time from randomization to the first of two negative cultures at different visits without an intervening positive culture result, irrespective of whether there were subsequent cultures positive for *Mycobacterium tuberculosis*, and time to smear negative status defined analogously. Cultures with contamination were excluded from all analyses and did not contribute to the definition of culture negative status. TTP over time was analyzed using a Bayesian non-linear mixed effects regression model as described previously, [17] and was summarized as the daily rate of change in log_10_(TTP) (bactericidal activity) from day 0 to day 56, BA(0–56), where TTP is measured in hours. The regression model implemented the specification of normally distributed residuals and random coefficients. Bacterial killing is often observed to be greater over the first 7–14 days of TB treatment [13]. However, since the earliest cultures in the REMoxTB trial were at 7 and 14 days, it was not reasonable to consider the early and late slopes separately and therefore BA(0–56) was chosen as the most appropriate measure to reflect the combination of both phases of killing. No culture results after treatment change or withdrawal from treatment were included in the analysis. Cultures after week 8 were also not included in the modelling of TTP over time to avoid undue influence in slope fitting of later positive culture results in the small number of patients that fail treatment and to more closely reflect a phase II endpoint.

Baseline predictors of outcome were evaluated using logistic regression separately within each treatment group and also with all patients combined, adjusted for treatment. Baseline covariates were evaluated firstly in univariable models and then in multivariable models if significant (at the 5 % level) on the likelihood ratio test. Baseline covariates tested were HIV status, presence of cavities on chest X-ray, history of smoking, sex, race, weight, body mass index (BMI), country and continent of study centre, smear grade, solid culture (LJ) grade, TTP on MGIT, CD4 count (HIV patients only) and resistance to isoniazid.

Trial-level surrogacy was evaluated by plotting differences between treatments on the marker of speed of clearance of bacilli with 95 % confidence interval against the differences on the primary endpoint. The primary efficacy outcome was the proportion of patients who had bacteriologically or clinically defined failure or relapse within 18 months following randomization (a composite unfavourable outcome). Negative culture status at 18 months (at or after 72 weeks) was considered a favourable outcome provided there was no prior unfavourable outcome and where the last positive culture result was followed by at least two negative culture results. The per protocol analysis population was used for this analysis, as this approach was closest to a pure bacteriological outcome of failure/relapse. The between-treatment difference in the probability of an unfavourable outcome was estimated from a generalized linear model with identity-link function adjusted for weight and study centre (as was done in the primary trial analysis). Differences between treatments with respect to time to culture or smear negative status were characterized using a hazard ratio from a Cox proportional hazards regression model. Full details of the primary trial analyses are given elsewhere [1].

Individual-level surrogacy was evaluated using the non-parametric Cuzick test for trend [18] on categorical variables and logistic regression to model the probability of an unfavourable clinical outcome. The continuous markers of speed of clearance of bacilli described above were included as independent variables using fractional polynomials [19] to allow for non-linear relationships. Time of last culture was used for the few patients who did not achieve culture or smear negative status (<5 % on MGIT, <2 % on LJ, <2 % smear). An alternative approach of using multiple imputation with upper limit censoring was used for the few patients that did not achieve culture negative status, but results were unchanged and so are not presented. The c-statistic [20], calculated as the area under the receiver operating characteristic curve (AUC_ROC_), was used to compare prediction models to identify the markers that had highest discrimination between high risk and low risk patients. Patients with missing values for the included baseline covariates were excluded from the covariate-adjusted AUC_ROC_ analysis.

### Ethical review

The ethics committee at University College London (London, UK) and all national and local ethics committees approved the trial, including these analyses which were planned as a secondary objective to the trial. All patients provided written or witnessed oral informed consent.

### Role of the funding source

The Global Alliance for TB Drug Development was involved in study design, data interpretation and writing of this report. All other funders were not involved in study design, data interpretation or writing of the report. The first author (PPJP) had full access to all the data in the study and had final responsibility for the decision to submit for publication.

### Availability of data

Raw data from the REMoxTB trial is available for eligible researchers as part of a repository of TB trial data. See http://c-path.org/programs/tb-pacts/ for further details.

## Results

### Baseline predictors

After adjusting for treatment arm, HIV co-infection, cavitation on X-ray, low BMI, history of smoking and male gender (Table 1) were significant predictors of an unfavourable outcome.

**Table 1:** Predictors of an unfavourable outcome for all data (adjusted for treatment) and within each treatment arm. Prediction models fitted all factors significant in the “all data” model (*p* <0.05, likelihood ratio test) with the addition of TTP on MGIT which was significant in the “ethambutol arm” model. Factors not listed in this table were not significant in any model

Multivariable odds ratio (95 % CI)	All data	Control arm patients only	Isoniazid arm patients only	Ethambutol arm patients only
Control	Reference			
Isoniazid	1.81 (1.18, 2.78)			
Ethambutol	2.88 (1.92, 4.33)			
	*p* <0.001			
BMI per 1 kg/m^2^	0.92 (0.87, 0.98)	0.93 (0.82, 1.05)	0.95 (0.85, 1.05)	0.90 (0.83, 0.99)
	*p* = 0.004	*p* = 0.193	*p* = 0.286	*p* = 0.027
History of smoking	1.63 (1.15, 2.31)	2.12 (0.99, 4.53)	2.34 (1.23, 4.46)	1.15 (0.69, 1.92)
	*p* = 0.005	*p* = 0.046	*p* = 0.007	*p* = 0.592
HIV positive	2.93 (1.69, 5.08)	2.64 (0.90, 7.78)	1.95 (0.72, 5.28)	4.46 (1.79, 11.09)
	*p* <0.001	*p* = 0.100	*p* = 0.210	*p* = 0.002
TTP on MGIT (per 0.1 log_10_(day))	0.97 (0.90, 1.05)	1.17 (1.03, 1.32)^a^	1.02 (0.89, 1.17)	0.84 (0.74, 0.95)
	*p* = 0.439	*p* = 0.024	*p* = 0.789	*p* = 0.003
Male gender	1.69 (1.13, 2.53)	1.10 (0.48, 2.55)	1.94 (0.94, 3.98)	2.09 (1.13, 3.85)
	*p* = 0.009	*p* = 0.817	*p* = 0.060	*p* = 0.014
Cavities on X-ray	1.93 (1.22, 3.05)	1.62 (0.62, 4.19)	2.82 (1.15, 6.94)	1.75 (0.89, 3.41)
	*p* = 0.003	*p* = 0.302	*p* = 0.013	*p* = 0.091

^a^This association (*p* = 0.024) indicates a higher probability of an unfavourable outcome with higher TTP on MGIT, indicating a lower bacillary load which is biologically counter-intuitive. This is a modest odds ratio with a fairly wide confidence interval—similar results are seen in the univariable model. Due to the multiplicity in the number of tests done to evaluate baseline predictors this is therefore likely a chance finding

### Trial-level surrogacy

**Fig. 1:**
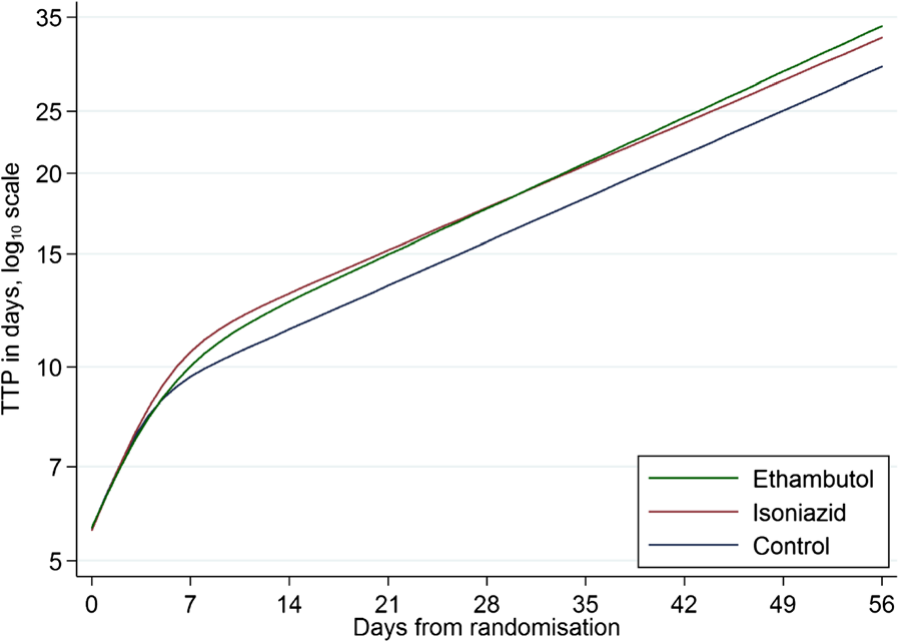
Fit of non-linear mixed effects model of MGIT TTP during the first 56 days of treatment with three anti-tuberculosis regimens

Culture negative status on both LJ and MGIT, but not smear negative status, was achieved earlier in both moxifloxacin arms as compared to the control. For time to culture negative status on MGIT the hazard ratio was 1.16, 95 % CI (1.02, 1.30) for both arms (log-rank *p* = 0.013 and *p* = 0.010 for the isoniazid and ethambutol arms, respectively) and for time to culture negative status on LJ the hazard ratio was 1.24, 95 % CI (1.10, 1.40), *p* <0.001 for the isoniazid arm and 1.20, 95 % CI (1.06, 1.35), *p* = 0.002 for the ethambutol arm. There was no reduction in time to smear negative status, hazard ratio 0.97, 95 % CI (0.86, 1.09), *p* = 0.503 for the isoniazid arm and 0.96, 95 % CI (0.85, 1.08), *p* = 0.611 for the ethambutol arm, compared to control. The daily rate of change in log_10_(TTP) over time was bi-phasic with a transition point before 14 days (Fig. 1). The rate of change in log_10_(TTP) from day 0 to day 56, BA(0–56), was higher in the ethambutol arm (0.0139 log_10_(hours) per day on treatment, 95 % Bayesian credibility interval (BCI) 0.0130, 0.0142) than in the control arm (0.0128, 95 % BCI 0.0123, 0.0134), difference 0.0010 (95 % BCI 0.0002, 0.0018). The rate of change in log_10_(TTP) was not higher in the isoniazid arm (0.0136 95 % BCI 0.0133, 0.0145) than in the control arm, difference 0.0008 (95 % BCI −0.0001, 0.0016).

**Fig. 2:**
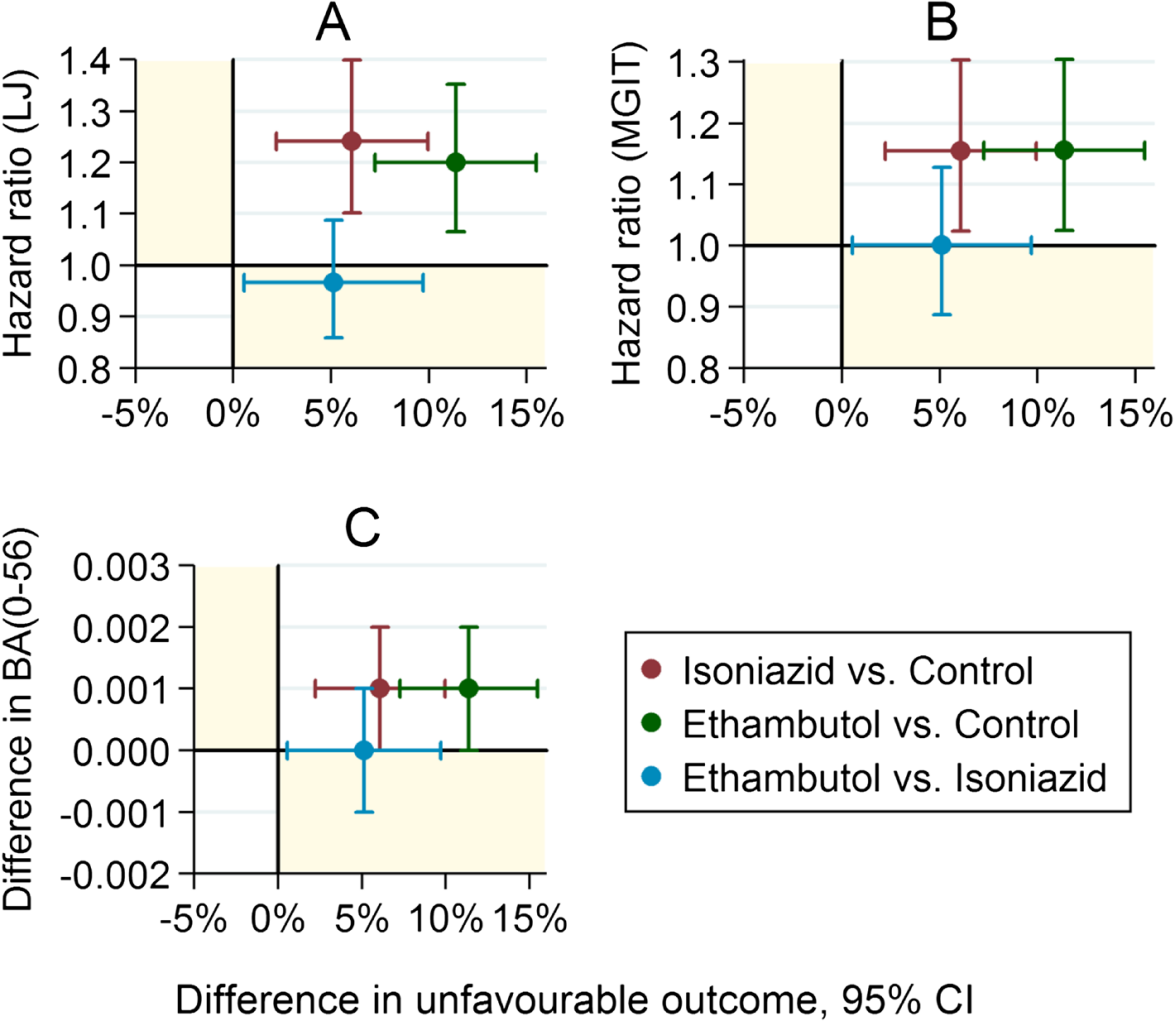
Trial-level surrogacy plot. **a** Time to culture negative status on LJ. **b** Time to culture negative status in MGIT. **c** BA(0–56), daily rate of change in log10(TTP) to day 56. The difference between treatments on the intermediate marker is plotted against the difference in unfavourable outcome with 95 % confidence intervals. Points lying outside the yellow regions indicate that the treatment difference is in the opposite direction on the intermediate marker from the long-term clinical outcome

Figure 2 shows the association between the difference between treatments on the markers of speed of clearance of bacilli and the difference between treatments on the long-term clinical outcome for the three culture-based markers. Each plotted point represents a single treatment comparison.

Although there is a modest benefit in both moxifloxacin arms with regard to each of the intermediate microbiological markers as compared to the control (the red and green points lie above the horizontal line of no difference), there are more unfavourable clinical outcomes (the red and green points lie to the right of the vertical line of no difference). The treatment effects on the intermediate and clinical outcomes are therefore in the opposite direction. Furthermore, although there is no significant difference with regard to any of the intermediate markers when comparing the two 4-month moxifloxacin arms, there is a higher proportion of unfavourable outcomes on the ethambutol arm. Similar results were seen for culture results at 6, 8 or 12 weeks (graphs not shown). Thus, trial-level surrogacy is not satisfied with any of these intermediate markers.

### Individual-level surrogacy

**Fig. 3:**
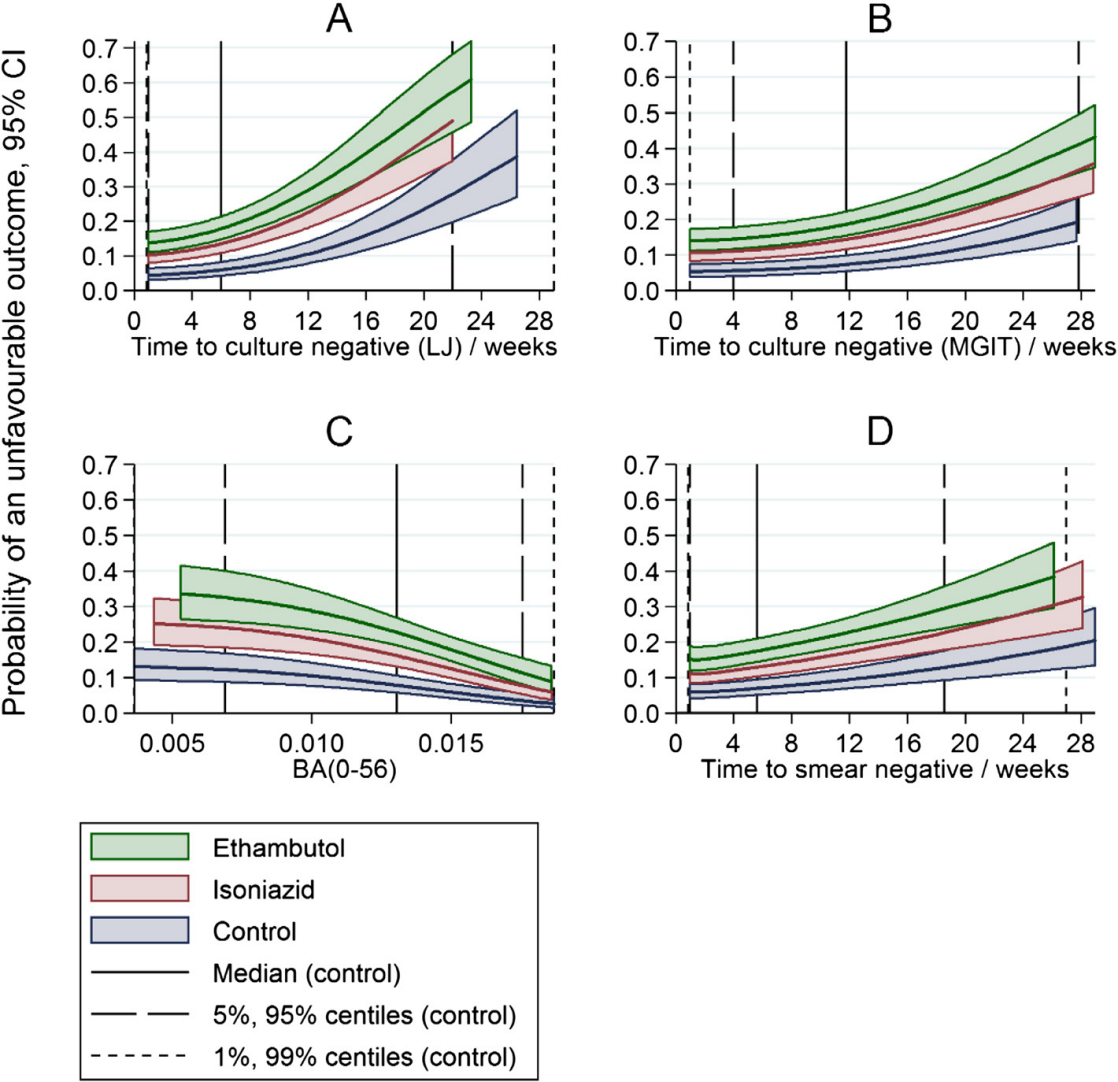
Estimates of probability of an unfavourable outcome by treatment arm and by intermediate marker. **a** Time to culture negative status on LJ. **b** Time to culture negative status in MGIT. **c** BA(0–56), daily rate of change in log_10_(TTP) to day 56. **d** Time to smear negative. Vertical solid and dashed lines show various centiles of the intermediate markers for patients in the control arm in the REMoxTB trial

Table 2 shows the number and proportion of patients with an unfavourable outcome at the end of follow-up, by categorical groupings of time to smear or culture negative status on LJ or on MGIT, or quartiles of BA(0–56). The proportion of patients with an unfavourable outcome is lower in those with faster clearance of bacilli (earlier smear or culture negative status achieved or a larger daily rate of change in log_10_(TTP)), *p* <0.001 in each case for arms grouped together. Considered as continuous, rather than categorical, all four intermediate markers are independent predictors of an unfavourable outcome, adjusted for treatment arm (Fig. 3, *p* <0.001). The curves are distinct and approximately parallel showing that the 6-month control regimen has better outcomes independently of speed of clearance of bacilli. However, the probability of an unfavourable outcome is non-zero for patients that achieve culture or smear negative status in the first few weeks as the curves reach non-zero asymptotes.

**Table 2:** Number of patients with an unfavourable outcome by treatment arm and groupings of time to culture negative status on LJ and MGIT. Groupings are quartiles or approximate quartiles for time to culture negative status

	Time	Control	Isoniazid	Ethambutol	Total
Grouping	n (%) / N	n (%) / N	n (%) / N	n (%) / N	n (%) / N
Time to culture negative status on LJ^a^	<4 weeks	8 (8 %) / 103	10 (9 %) / 106	14 (11 %) / 122	32 (10 %) / 331
	4 to <6 weeks	7 (6 %) / 117	13 (9 %) / 139	19 (16 %) / 117	39 (10 %) / 373
	6 to <8 weeks	6 (4 %) / 134	16 (12 %) / 132	33 (20 %) / 161	55 (13 %) / 427
	8+ weeks	19 (12 %) / 153	37 (27 %) / 135	36 (30 %) / 121	92 (22 %) / 409
	Total	40 (8 %) / 507	76 (15 %) / 512	102 (20 %) / 521	218 (14 %) / 1,540
	Test for trend	*p* = 0.275	*p* <0.001	*p* <0.001	*p* <0.001
Time to culture negative status in MGIT^b^	<6 weeks	4 (5 %) / 85	9 (8 %) / 109	12 (11 %) / 110	25 (8 %) / 304
	6 to <8 weeks	4 (4 %) / 99	12 (10 %) / 116	15 (13 %) / 120	31 (9 %) / 335
	8 to <12 weeks	3 (4 %) / 74	11 (14 %) / 76	14 (19 %) / 74	28 (13 %) / 224
	12+ weeks	29 (12 %) / 249	43 (20 %) / 210	58 (27 %) / 214	130 (19 %) / 673
	Total	40 (8 %) / 507	75 (15 %) / 511	99 (19 %) / 518	214 (14 %) / 1,536
	Test for trend	*p* = 0.013	*p* = 0.002	*p* <0.001	*p* <0.001
BA(0–56), daily rate of change in log_10_(TTP) to day 56^c^	<0.01153	17 (10 %) / 165	22 (19 %) / 116	30 (31 %) / 98	69 (18 %) / 379
	0.01153 to <0.0137	11 (9 %) / 119	27 (21 %) / 130	30 (23 %) / 131	68 (18 %) / 380
	0.0137 to <0.01581	7 (6 %) / 111	15 (11 %) / 133	24 (18 %) / 135	46 (12 %) / 379
	>0.01581	4 (4 %) / 104	11 (9 %) / 127	20 (13 %) / 149	35 (9 %) / 380
	Total	39 (8 %) / 499	75 (15 %) / 506	104 (20 %) / 513	218 (14 %) / 1,518
	Test for trend	*p* = 0.040	*p* = 0.004	*p* = 0.001	*p* <0.001
Time to smear negative status^d^	<4 weeks	11 (8 %) / 134	11 (7 %) / 154	21 (17 %) / 127	43 (10 %) / 415
	4 to <6 weeks	10 (8 %) / 122	13 (13 %) / 101	17 (15 %) / 115	40 (12 %) / 338
	6 to <8 weeks	4 (4 %) / 93	14 (17 %) / 82	15 (16 %) / 95	33 (12 %) / 270
	8+ weeks	15 (9 %) / 158	37 (21 %) / 173	50 (27 %) / 185	102 (20 %) / 516
	Total	40 (8 %) / 507	75 (15 %) / 510	103 (20 %) / 522	218 (14 %) / 1,539
	Test for trend	*p* = 0.926	*p* <0.001	*p* = 0.026	*p* <0.001

^a^Excluding 8 patients censored before time to culture negative status before 8 weeks; ^b^excluding 12 patients censored before time to culture negative status before 12 weeks; ^c^excluding 30 patients with insufficient data to be included in model; ^d^excluding 9 patients censored before time to culture negative status before 8 weeks. n, number of patients with an unfavourable outcome; N, number of assessable patients; %, number of patients with an unfavourable outcome relative to the number of assessable patients

While in a univariable model baseline TTP is a predictor of outcome, after adjusting for treatment arm and intermediate marker, baseline TTP was not an independent predictor of outcome, *p* = 0.77 and *p* = 0.75 for time to culture negative status on LJ and MGIT, respectively, *p* = 0.09 for BA(0–56) and *p* = 0.68 for time to smear negative status.

### Comparing models

**Fig. 4:**
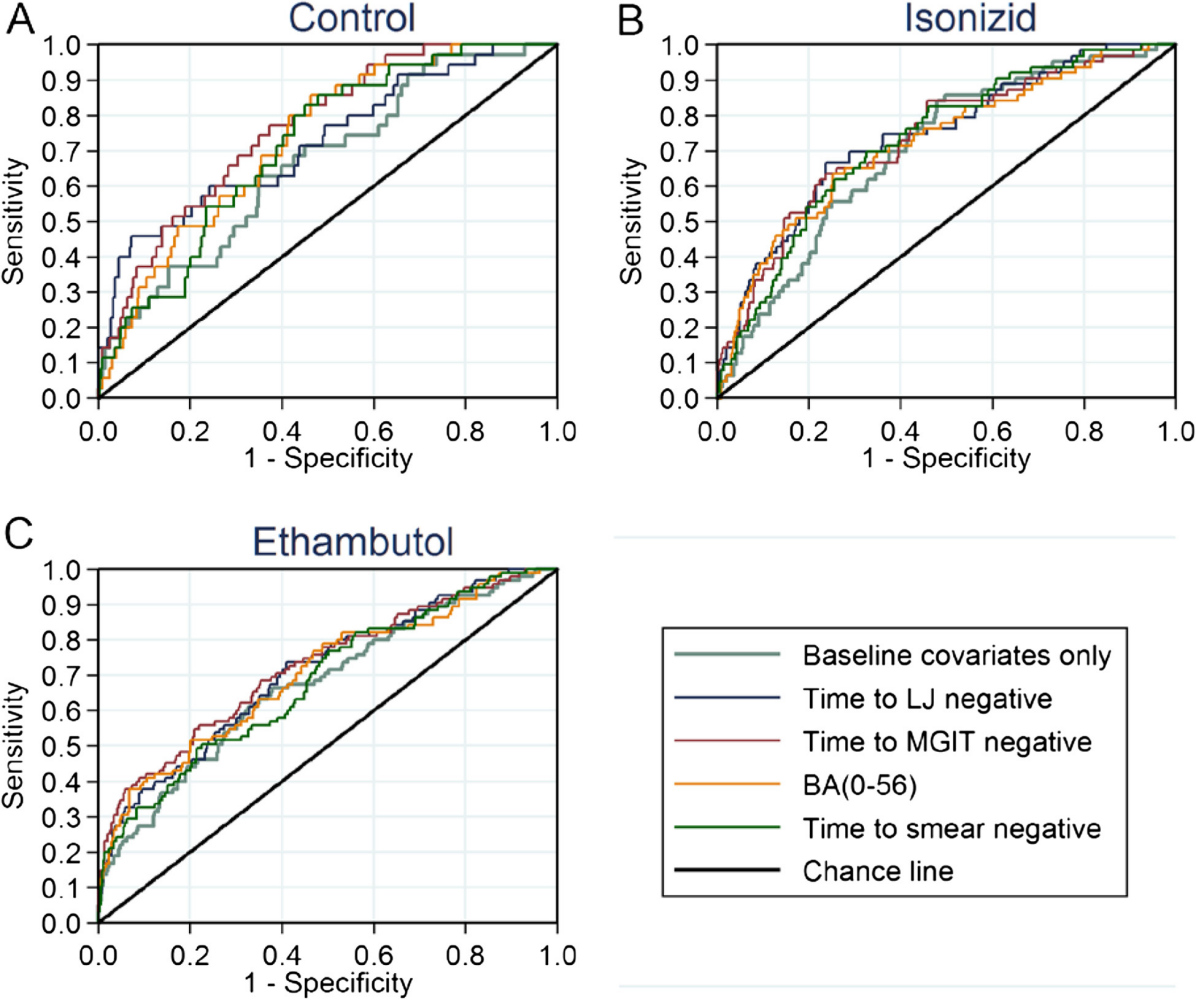
Receiver operating characteristic (ROC) curves. All curves represent models adjusted for baseline covariates. **a** Control arm. **b** Isoniaizid arm. **c** Ethambutol arm

Table 3 shows the AUC_ROC_ for each of the markers demonstrating the ability of the model to discriminate unfavourable from favourable outcomes. Although the confidence intervals around the estimates are fairly wide, the estimates of AUC were higher for time to culture negative status, BA(0–56) and time to smear than culture or smear results at a single visit indicating better discrimination. Discrimination was improved on adjusting for baseline covariates. However, none of the markers resulted in greatly improved discrimination over the reference model adjusted for baseline covariates with the greatest improvements seen in the control arm. Figure 4 shows ROC curves for a selection of markers.

**Table 3:** Table of area under the receiver operating characteristic curve (AUC_ROC_) and 95 % confidence intervals for various models. Baseline covariates fitted in the adjusted models include those found to be significant in Table 2: BMI, history of smoking, HIV status, gender, presence of cavitation and baseline DTP in MGIT

	Control	Isoniazid		Ethambutol		Combined (adjusted for treatment)		
	Unadjusted	Adjusted for baseline covariates	Unadjusted	Adjusted for baseline covariates	Unadjusted	Adjusted for baseline covariates	Unadjusted	Adjusted for baseline covariates
No on-treatment predictors (reference)	0.50	0.67 (0.57, 0.76)	0.50	0.70 (0.64, 0.77)	0.50	0.67 (0.61, 0.74)	0.60 (0.57, 0.64)	0.70 (0.66, 0.74)
BA(0–56), daily rate of change in log10(TTP) to day 56	0.60 (0.52, 0.69)	0.73 (0.66, 0.81)	0.61 (0.55, 0.68)	0.72 (0.65, 0.79)	0.61 (0.55, 0.67)	0.70 (0.64, 0.76)	0.66 (0.62, 0.70)	0.73 (0.69, 0.76)
Time to culture negative status on LJ	0.61 (0.50, 0.72)	0.73 (0.63, 0.82)	0.67 (0.60, 0.74)	0.74 (0.67, 0.81)	0.63 (0.57, 0.69)	0.71 (0.65, 0.77)	0.70 (0.66, 0.74)	0.74 (0.70, 0.78)
Time to culture negative status in MGIT	0.59 (0.49, 0.70)	0.77 (0.69, 0.84)	0.62 (0.55, 0.69)	0.74 (0.67, 0.80)	0.64 (0.57, 0.70)	0.72 (0.66, 0.78)	0.67 (0.63, 0.70)	0.74 (0.70, 0.78)
Time to smear negative status	0.62 (0.51, 0.72)	0.72 (0.64, 0.79)	0.62 (0.56, 0.69)	0.73 (0.67, 0.79)	0.59 (0.53, 0.66)	0.68 (0.62, 0.74)	0.66 (0.62, 0.70)	0.72 (0.68, 0.76)
Week 6 culture on LJ	0.55 (0.47, 0.63)	0.68 (0.60, 0.75)	0.56 (0.50, 0.62)	0.70 (0.63, 0.77)	0.56 (0.51, 0.61)	0.68 (0.61, 0.74)	0.63 (0.59, 0.67)	0.71 (0.67, 0.75)
Week 8 culture on LJ	0.51 (0.45, 0.56)	0.66 (0.57, 0.74)	0.56 (0.51, 0.61)	0.71 (0.64, 0.77)	0.56 (0.52, 0.60)	0.69 (0.63, 0.75)	0.63 (0.59, 0.66)	0.71 (0.66, 0.75)
Week 12 culture on LJ	0.55 (0.50, 0.60)	0.71 (0.63, 0.80)	0.51 (0.49, 0.53)	0.69 (0.63, 0.76)	0.53 (0.50, 0.55)	0.69 (0.63, 0.75)	0.62 (0.59, 0.66)	0.71 (0.67, 0.75)
Week 6 culture in MGIT	0.49 (0.41, 0.57)	0.65 (0.55, 0.74)	0.51 (0.45, 0.57)	0.70 (0.63, 0.76)	0.54 (0.49, 0.60)	0.65 (0.59, 0.72)	0.61 (0.57, 0.64)	0.70 (0.66, 0.74)
Week 8 culture in MGIT	0.56 (0.48, 0.64)	0.70 (0.61, 0.79)	0.57 (0.51, 0.63)	0.71 (0.64, 0.78)	0.61 (0.55, 0.66)	0.70 (0.64, 0.76)	0.64 (0.60, 0.68)	0.72 (0.68, 0.76)
Week 12 culture in MGIT	0.58 (0.51, 0.65)	0.72 (0.64, 0.80)	0.54 (0.49, 0.58)	0.71 (0.64, 0.77)	0.55 (0.51, 0.59)	0.69 (0.63, 0.75)	0.63 (0.59, 0.67)	0.72 (0.68, 0.76)
Week 6 smear	0.49 (0.41, 0.57)	0.65 (0.56, 0.75)	0.53 (0.47, 0.59)	0.70 (0.63, 0.76)	0.57 (0.52, 0.63)	0.68 (0.62, 0.75)	0.62 (0.58, 0.66)	0.71 (0.67, 0.75)
Week 8 smear	0.53 (0.46, 0.61)	0.68 (0.59, 0.77)	0.55 (0.49, 0.61)	0.71 (0.64, 0.77)	0.57 (0.52, 0.62)	0.69 (0.63, 0.75)	0.63 (0.59, 0.67)	0.71 (0.67, 0.75)
Week 12 smear	0.54 (0.48, 0.61)	0.68 (0.60, 0.77)	0.55 (0.50, 0.60)	0.70 (0.64, 0.77)	0.58 (0.53, 0.62)	0.68 (0.62, 0.74)	0.63 (0.60, 0.67)	0.71 (0.67, 0.75)

## Discussion

Our data show that while various measures of speed of clearance of bacilli are predictors of clinical outcome, the ability of each marker to actually discriminate between favourable and unfavourable status is poor. Time to culture negative status on LJ and in MGIT, time to smear negative status and the daily rate of change of log_10_(TTP) in MGIT over 56 days tended to have higher discrimination as predictors than a culture or smear result at a single visit. Adjusting only for the baseline covariates, with no on-treatment information, AUCs ranged from 0.67 to 0.70 showing that each of these intermediate markers only modestly improved the prediction of an unfavourable outcome when important risk factors are known, including HIV status, presence of cavities, BMI and smoking history. In comparison, a recent study in 35 patients assessed various positron emission tomography/computed tomography (PET/CT) imaging biomarkers with AUC_ROC_ upwards of 0.9, although the authors acknowledge that this was a preliminary, hypothesis-generating analysis with small patient numbers [21].

An important finding in this work is that we demonstrated that there is a small but non-negligible proportion of patients who clear bacilli quickly but have a poor long-term bacteriological outcome on all three arms. This means that there are mechanisms of relapse that are not captured by these culture-based intermediate markers which only measure viable bacilli. This may be because the sub-population of bacteria that go on to cause relapse are lipid-rich, non-culturable persisters [6, 22, 23] that undergo transcriptional adaptation [24] or are not expectorated in sputum [21].

We found that time to smear negative status was a predictor of clinical outcome, although there was no difference in the effect of treatment on this endpoint indicating that it is unsuitable as a primary endpoint for a trial, in contrast to the faster time to culture negative status seen in the moxifloxacin regimens. This is consistent with the poor sensitivity of smear for predicting outcome [7] and makes it unlikely to be a useful marker for evaluating novel regimens.

The bi-phasic increase in log_10_(TTP) over time was consistent with other studies and the estimate of the rate of change in log_10_(TTP) of 0.013 in the control arm was consistent with another recently published study where the estimate was 0.017 [14].

TTP on MGIT at baseline, an established marker of bacterial load, was not an independent predictor of outcome after adjusting for these factors. Relapse rates have been observed to differ between patients from Asia and patients from Africa [16], but geographical region was also not an independent predictor of outcome in this study. These results indicate that patient factors and cavitation are more important than bacillary load as risk factors for a poor outcome of treatment.

In predicting the outcome for an individual patient, delayed culture conversion is associated with an increased risk of an unfavourable clinical outcome, but discrimination is modest. Even on the ethambutol regimen which had the poorest results, the majority of patients who had not achieved culture negative status on MGIT by 12 weeks (63 %) or who had not achieved culture negative status on LJ by 8 weeks (70 %) still went on to have a favourable outcome. This shows the limitations in using these markers in individual patient care.

There were some limitations in our study. We excluded results from contaminated cultures from all analyses, although these results might be informative for prediction models. A thorough analysis of surrogate endpoints should include multiple treatment comparisons of drugs with different mechanisms of action from multiple trials. Unfortunately REMoxTB is the only TB phase III trial of novel regimens to date with sufficiently frequent cultures during treatment to allow an assessment of time to culture conversion and daily rate of change of log_10_(TTP) in MGIT to day 56 as putative surrogate endpoints. As more trial data becomes available, these analyses will be updated. In addition, we were unable to definitively evaluate any of these markers as trial-level surrogates due to the differences between regimens in the continuation phase of treatment. This will be a failing of any putative surrogate endpoint that is measured before the end of treatment, as it will not be able to fully capture the treatment effect. However, the comparison of the 4-month regimens showed that, even when the duration of treatment is the same, while there was no difference in speed of clearance of bacilli, there were more unfavourable outcomes on the ethambutol arm. This observation suggests two explanations. None of the drugs being compared between regimens have traditionally been thought to have strong sterilizing activity and it may therefore be that trial-level surrogacy may be satisfied in an evaluation of a regimen with a stronger sterilizing effect such as one with an increased dose of rifampicin. Nevertheless, a surrogate endpoint that is dependent on the regimens under comparison will only be of limited use in drug development decision-making. Alternatively, these results may show that the addition of isoniazid in the continuation phase of treatment does help prevent relapse, which would support the important role of isoniazid as a drug with both bactericidal and sterilizing activity [25].

The primary endpoint of the REMoxTB trial was a composite outcome including relapse and failure. The majority of outcomes in the per protocol population were confirmed by bacteriology, but a limitation of this analysis is that a small number of outcomes may not represent true treatment failures or relapses. Nevertheless, this endpoint is the accepted endpoint for pivotal TB phase III trials and is therefore most relevant for this surrogacy analysis.

We welcome a recent model using the proportion of patients that are culture positive at 2 months on LJ to predict phase III outcomes [26], which performs fairly well in a retrospective analysis using the results of intermediate outcomes from the large phase III trials [27]. The prediction intervals are, however, wide (80 % intervals are presented). The variability in the proportion remaining culture positive after 2 months observed in small phase II trials (20 % [28], 29 % [29] and 18 % [13] for the ethambutol-sparing moxifloxacin regimen and 1 % to 21 % [16] for the well-studied combination of daily streptomycin, rifampicin, isoniazid and pyrazinamide) means that the precision in predicting phase III trial results prospectively from phase II results is likely to be low.

The modest benefits with the addition of a fluoroquinolone seen in pre-clinical and early-phase clinical trials did not enable treatment to be shortened from 6 to 4 months [30–32]. It is unclear how much larger the effects from novel regimens would need to be in order to permit treatment-shortening, but we have shown that markers that are better individual- and trial-level surrogates are also needed—preferably measured at the end of treatment—to give greater confidence in moving novel regimens to expensive phase III trials. Moreover, the mechanism underlying the poor outcome in some patients who cleared their infection rapidly from sputum requires further investigation. Until improved markers are available, culture-based markers will be the primary endpoints in the middle phase of clinical development, but results from these clinical trials should be interpreted with caution. Innovative clinical trial designs may also have a role in managing the risk in moving between phases of clinical trials [33, 34].

## Conclusions

In summary, we have shown that culture conversion during treatment for tuberculosis has only a limited role in decision-making for advancing novel regimens into pivotal phase III clinical trials or in predicting the outcome of treatment for individual patients.
