# Erratum to: Limited role of culture conversion for decision-making in individual patient care and for advancing novel regimens to confirmatory clinical trials

**DOI:** 10.1186/s12916-016-0585-7

**Published:** 2016-02-23

**Authors:** Patrick P. J. Phillips, Carl M. Mendel, Divan A. Burger, Angela M. Crook, Andrew J. Nunn, Rodney Dawson, Andreas H. Diacon, Stephen H. Gillespie

**Affiliations:** MRC Clinical Trials Unit at UCL, Aviation House, 125 Kingsway, London, WC2B 6NH UK; Global Alliance for TB Drug Development, New York, NY USA; Department of Mathematical Statistics and Actuarial Science, University of the Free State, Bloemfontein, South Africa; Division of Pulmonology and Department of Medicine, University of Cape Town Lung Institute, Mowbray, Cape Town South Africa; Division of Physiology, Department of Medical Biochemistry, Stellenbosch University, Tygerberg, Cape Town South Africa; TASK Applied Science, Bellville, Cape Town South Africa; School of Medicine, University of St Andrews, St Andrews, UK

## Erratum

The original version of this article [1] unfortunately contained a mistake. In the author list, the middle initial of author Angela M. Crook was missing. The original article has also been updated with the correct spelling.
